# P55 Attempting to reduce attendance in the Emergency Department for patients requiring antibiotics who could have gone to their GP—a Health Equity approach

**DOI:** 10.1093/jacamr/dlag102.061

**Published:** 2026-06-26

**Authors:** K Atack, G Sheerman-Chase, B Effendi, G Powell, M Cutts

**Affiliations:** Medicines Management and Pharmacy Services, Leeds Teaching Hospitals NHS Trust, UK; West Yorkshire Integrated Care Board, UK; West Yorkshire Integrated Care Board, UK; West Yorkshire Integrated Care Board, UK; Leeds City Council, Leeds, UK

## Abstract

**Objectives:**

Identify whether patients who live near St. James’s hospital (in postcode LS9) are attending the Emergency Department (ED) for antibiotics rather than visiting primary care. The area around the hospital has a high migrant population and has a high index of social deprivation. Identify the proportion of patients attending ED who were prescribed antibiotics that they could have obtained in primary care. Identify diversity of ethnic backgrounds of these patients. Identify the proportion of patients who required an interpreter.

**Methods:**

Data from 218 patients who received oral antibiotics and lived near ED versus 218 patients who live across place as analysed. The medical notes were reviewed and assessed to identify whether it would have been more appropriate for patients to have accessed treatment for their infection in primary care. Demographics including demographics including ethnic background, age, gender and interpreter need were analysed.

**Results:**

22% of patients who lived in LS9 could have obtained antibiotics from primary care versus 27% from across place. 49% of LS9 patients could have obtained antibiotics from primary care were non-white British versus 55% from across place. 16% of LS9 patients could have obtained antibiotics from primary care required a translator versus 4% from across place.

**Conclusions:**

Relative to the whole sample, there was a significant proportion of non-white people who attended ED for antibiotics that they could have obtained in primary care. Interestingly, attendance was similar across the whole place. It should be noted that a higher proportion of LS9 patients who could have attended primary care required a translator. This is understandable given the population around this area, where English is more likely to be a second language for these groups. Patients need to be provided with information about which primary care services to attend to obtain antibiotics and reduce the number of unnecessary attendances to the Emergency Department. Information should be appropriate, accessible and in multiple languages. A collaboration between primary care, the council and hospital has been established to improve education and resources and have been developed in a number of languages to give to communities to reduce unnecessary attendance to ED and improve accessibility. These resources are posters, leaflets and videos and have been distributed throughout the LS9 community, but there are also plans to have these resources in ED for patients to access and clinicians to provide to them. Once the resources have been disseminated there is a plan to review a proportion of attendances to ED to identify whether this campaign has had any impact.

